# Nutritional Potential of Adzuki Bean Germplasm and Mining Nutri-Dense Accessions through Multivariate Analysis

**DOI:** 10.3390/foods12224159

**Published:** 2023-11-17

**Authors:** Deepika D. D., Siddhant Ranjan Padhi, Padmavati G. Gore, Kuldeep Tripathi, Ashvinkumar Katral, Rahul Chandora, G. J. Abhishek, Vishal Kondal, Rakesh Singh, Rakesh Bharadwaj, Kailash C. Bhatt, Jai Chand Rana, Amritbir Riar

**Affiliations:** 1 The Graduate School, ICAR-Indian Agricultural Research Institute, PUSA, New Delhi 110012, India; dddeepika.dd@gmail.com (D.D.D.); siddhant.padhi1@gmail.com (S.R.P.); ashokgkatral@gmail.com (A.K.); golsarabhishek50@gmail.com (G.J.A.); 2 ICAR—National Bureau of Plant Genetic Resource, Pusa, New Delhi 110012, India; padmavati.gore@icar.gov.in (P.G.G.); kuldeep.tripathi@icar.gov.in (K.T.); rahul1@icar.gov.in (R.C.); vishalkondal4@gmail.com (V.K.); rakesh.singh2@icar.gov.in (R.S.); 3 Division of Plant Exploration and Germplasm Collection, ICAR—National Bureau of Plant Genetic Resources, Pusa, New Delhi 110012, India; 4 The Alliance of Bioversity International & CIAT—India Office, New Delhi 110012, India; j.rana@cgiar.org; 5 Department of International Cooperation, Research Institute of Organic Agriculture FiBL, 5070 Frick, Switzerland; amritbir.riar@fibl.org

**Keywords:** underutilized legume, nutritional composition, variability, multivariate analysis, ANOVA

## Abstract

The adzuki bean (*Vigna angularis*), known for its rich nutritional composition, holds significant promise in addressing food and nutritional security, particularly for low socioeconomic classes and the predominantly vegetarian and vegan populations worldwide. In this study, we assessed a total of 100 diverse adzuki bean accessions, analyzing essential nutritional compounds using AOAC’s official analysis procedures and other widely accepted standard techniques. Our analysis of variance revealed significant genotype variations for all the traits studied. The variability range among different traits was as follows: moisture: 7.5–13.3 g/100 g, ash: 1.8–4.2 g/100 g, protein: 18.0–23.9 g/100 g, starch: 31.0–43.9 g/100 g, total soluble sugar: 3.0–8.2 g/100 g, phytic acid: 0.65–1.43 g/100 g, phenol: 0.01–0.59 g/100 g, antioxidant: 11.4–19.7 mg/100 g GAE. Noteworthy accessions included IC341955 and EC15256, exhibiting very high protein content, while IC341957 and IC341955 showed increased antioxidant activity. To understand intertrait relationships, we computed correlation coefficients between the traits. Principal Component Analysis (PCA) revealed that the first four principal components contributed to 63.6% of the variation. Further, hierarchical cluster analysis (HCA) identified nutri-dense accessions, such as IC360533, characterized by high ash (>4.2 g/100 g) and protein (>23.4 g/100 g) content and low phytic acid (0.652 g/100 g). These promising compositions provide practical support for the development of high-value food and feed varieties using effective breeding strategies, ultimately contributing to improved global food security.

## 1. Introduction

Legumes are essential for meeting the dietary and nutritional needs of both people and animals. The global population is projected to increase by 170% by 2050, posing enormous challenges to the global community in ensuring secure and safe food supplies [1]. Legumes represent a unique food crop group aligning with sustainable goals while providing a substantial amount of essential nutrients. Adzuki beans, in particular, stand out as highly resilient crops, showcasing remarkable adaptability to various growing conditions in both temperate and semitropical areas. They demonstrate a capacity to thrive even in nutrient-deficient soils. Given their seeds’ high-quality protein content, low carbon and water footprint, shorter growth period, ability to improve soil fertility through nitrogen fixation, and yield well in marginal lands, adzuki beans have the potential to significantly contribute to attaining sustainable development goals (SDGs), notably, SDG 2 (zero hunger), SDG 3 (good health and well-being), and SDG 13 (climate action). The adzuki bean (*Vigna angularis* (Willd) Ohwi and Ohashi) belonging to the Fabaceae family with a chromosome number of 2n = 2X = 22 holds immense potential to serve as a mainstream legume crop. The precise location of adzuki bean domestication remains uncertain, with suggestions of multiple domestication origins in northeast Asia, including Japan, China, and Korea [2,3,4,5]. *Vigna angularis* var. *nipponeinsis* (Ohwi) Ohwi and H. Ohashi (2n = 22), locally known as Ta-phe in East Kameng (Arunachal Pradesh, India) and reported to distribute in the North-Eastern Hill (NEH) region, is considered a close progenitor of cultivated adzuki beans. The adzuki bean provides an affordable, low-cost, easily digestible, rich source of protein and also contains carbohydrates, vitamins (A, B1, B2, B3, B9, and C), dietary fibers, and minerals (zinc, calcium, and iron). However, it also contains anti-nutritional factors like phytates, α-galactosides, and trypsin inhibitors with varying concentrations in different cultivars of adzuki beans [6]. The amino acid profile of adzuki bean protein is appropriately balanced, meeting the criteria set forth by the World Health Organization (WHO) and the Food and Agriculture Organization (FAO) [7]. An analysis of different varieties of adzuki beans was carried out to examine their amino acid composition. The findings showed that all eight essential amino acids needed for human nutrition were present in all the varieties. Notably, these adzuki beans had higher levels of leucine, lysine, valine, and phenylalanine. Lysine content, in particular, stood out, ranging from 1.98 to 1.82 g/100 g of beans. These amino acids are typically deficient in cereal proteins, emphasizing the unique nutritional value provided by them [8].

The adzuki bean is an environment-friendly legume that can fix atmospheric nitrogen, enhance soil fertility, and prevent soil erosion. It is a multipurpose legume crop where the young pods are used as vegetables, grains as pulses, fodder, and green manure. *V*. *angularis* seems to have the highest level of cold tolerance among cultivated Vigna, based on its geographical distribution in the temperate zone [9]. Compared to other grain legumes, adzuki beans are more resistant to heavy rains and can thrive in all types of soil. With over 670,000 ha under cultivation and a production of 2,601,000 tons, China was the world’s largest producer of adzuki beans in 2019–2020, followed by Japan at 140,000 tons/year [10]. The adzuki bean offers several health benefits; its grains are a good source of folate. They are easy to cook, possess a delicious taste, are good for weight management, slow down blood sugar, and are easy to digest. Adzuki-bean-derived food products may lower blood cholesterol, while proteins and phenols present in them act as antidiabetic agents [11,12]. Adzuki bean sprouts have been consumed as food and herb medicine by Chinese folk to maintain health and regulate weight since the Tang Dynasty [13,14,15]. The antioxidants and other bioactive components found in the adzuki bean seed coat are reported to lessen stress and inflammation during hypertension, supporting good health and attracting significant attention [16,17]. According to Fukuda et al. [18], adzuki bean consumption is linked to a lower risk of lifestyle-related diseases in people. Adzuki bean cultivars showed different antioxidant activities and cytoprotective effects according to the concentration and composition of phenolic compounds [19,20,21].

Owing to such characteristics, the adzuki bean is widely used in the preparation of a variety of foods, often sweetened before eating [22]. In particular, it is boiled with sugar, resulting in red bean paste (anko). Red bean paste is used in many Chinese dishes and also serves as a filling in Japanese sweets such as anpan, dorayaki, daifuku, etc. In Japan, China, and Korea, they are consumed daily as a dessert or snack and at celebrations and traditional festivals. Ignorance about the potentials of the adzuki bean and lack of information on the extent of genetic variations within the crop are major bottlenecks for cultivar development and popularizing it as a mainstream pulse crop despite its high nutritional quality [2]. Characterization and evaluation data, particularly nutrient composition data, will support the selection of nutri-dense accessions and also promote and popularize this legume crop. There are limited reports available on the nutritional constituents of adzuki beans from India. Hence, the present investigation has been undertaken to explore the nutritional compositions of exotic and indigenous adzuki bean collections from the National Gene Bank, ICAR-NBPGR, New Delhi, India.

## 2. Materials and Methods

### 2.1. Adzuki Bean Seed Material

A total of 100 adzuki bean accessions, comprising both exotic and indigenous collections, along with three checks of adzuki beans, were carefully selected from the National Gene Bank, ICAR- National Bureau of Plant Genetic Resources (ICAR-NBPGR), New Delhi, India (Table S1). These selected accessions were sown during the *Kharif* season of 2021 at the experimental fields of the ICAR- NBPGR, Regional Station Shimla, India. The experimental station is situated in the humid temperate zones of India, positioned at a latitude of 31.1048° N and a longitude of 77.1734° E. The station’s altitude is 2276 m AMSL, and it receives an annual rainfall of approximately 1415 mm. All the accessions were grown under natural conditions. Each accession was grown in a single row with two replications, following a randomized complete block design (RCBD). The rows had a bed length of 2 m, a plant-to-plant spacing of 20 cm, and a row-to-row spacing of 60 cm. Standard prescribed agricultural practices were adhered to, from sowing to harvesting. During field preparation, 100 kg/ha of di-ammonium phosphate (DAP) was incorporated. Irrigation was provided pre-sowing to ensure proper germination and additional irrigation was given at the blooming stage. Care was taken to maintain the required soil moisture through a suitable irrigation schedule. Weeding was carried out 25–30 days after sowing. No crop protection chemicals were sprayed throughout the entire crop cycle.

At the physiological maturity stage, adzuki bean pods were harvested. Pods from each row were harvested separately and sun-dried, and the seeds were extracted. Each row’s harvest constituted one replicate, resulting in two replicates per accession. The evaluation of nutritional composition for the adzuki bean germplasm was conducted during the year 2021–2022 at the Division of Germplasm Evaluation at ICAR-NBPGR, New Delhi, India.

### 2.2. Nutrional Composition Analysis

#### 2.2.1. Total Moisture Content

The moisture content was determined following the AOAC 925.09 [23] method, with a slight modification of considering the hot weight. Porcelain crucibles and their lids were initially dried for an hour at 100 °C in an air oven, and the hot weight of the empty crucible was recorded. Then, 3 g of adzuki flour was accurately weighed in duplicate and placed into a pre-weighed, dried crucible. The crucible with the sample was then placed in a 100 ± 5 °C air oven for 2–3 h and reweighed until a constant weight was achieved.

#### 2.2.2. Total Ash Content

The total ash content was determined using the AOAC 923.03 [23] method. An empty crucible was marked and heated in a furnace at 100 °C for 1 h. The weight of the hot empty crucible was noted. Subsequently, 3 g of the sample, in duplicate, was added to the pre-weighed crucible. The crucible with the sample was subjected to 180 °C for 1 h in the furnace, followed by an increase to 250 °C for 1 h to char the sample. The temperature was then gradually increased to 450 °C at a rate of approximately 20 °C per minute and maintained at 450 °C for 3 h. After complete ashing, the hot crucibles were transferred to a hot air oven maintained at 100 °C and left overnight to cool down. The weight of the crucible at 100 °C was recorded, and the ash content was calculated.

#### 2.2.3. Total Protein Content

The DUMAS method AOAC 992.23 [23] was utilized to estimate the total nitrogen concentration, employing the Elementar Rapid MAX N Exceed nitrogen auto analyzer. Approximately one hundred milligrams of the sample were carefully weighed in a stainless steel crucible using a Sartorius Electronic weighing balance (Model ATX224R), which was connected to the DUMAS instrument. The sample crucibles were then placed in a 90-place autosampler. Subsequently, the samples underwent combustion at 950 °C in a high oxygen environment, where nitrogen oxides were converted to nitrogen employing a copper–platinum catalyst and estimated using a thermal conductivity detector. The instrument was calibrated using HPLC grade L-Aspartic acid (Sigma A9256-100G Sigma-Aldrich, Saint Louis, MO, USA)) for nitrogen recovery. The nitrogen concentration was translated to a protein percentage by applying Jones’s factor of 6.25.

#### 2.2.4. Total Starch Content

Approximately 100 mg of homogenized sample underwent double extraction with 80% ethanol for 30 min at 80 °C. After each extraction cycle, the samples were centrifuged at 10,000 rpm for 10 min, the supernatants were collected, and the resulting residue was used to calculate the total starch content in the samples. Megazyme’s total starch assay kit, in accordance with AOAC 996.11 [23], was employed for this purpose. Initially, α-amylase was used to hydrolyze starch into maltodextrin, followed by amyloglucosidase to further hydrolyze maltodextrin into D-glucose. The oxidation of glucose led to the liberation of hydrogen peroxide, which was detected using a GOPOD reagent, producing a pink color. Absorbance was measured at 510 nm using a UV–vis spectrophotometer, BenchTop Lab Systems, Saint Louis, MO, USA and the results were expressed as g/100 g.

#### 2.2.5. Total Soluble Sugar (TSS) Content

For TSS analysis, the extraction was carried out using the same protocol as outlined in the starch section. TSS content was determined using the anthrone reagent method [24], involving sugar dehydration by sulfuric acid to generate furfural. The absorbance of the furfural anthrone complex (blue-green) at 630 nm was measured using a UV–vis spectrophotometer, and a calibration curve was established using a D-glucose standard. This technique quantifies the amount of TSS, including mono-, di-, and oligosaccharides, and the results were presented as glucose equivalents in g/100 g.

#### 2.2.6. Total Phytic Acid Content

The Phytate assay kit (K-PHYT) from Megazyme International, Wicklow, Ireland, was utilized to determine phytic acid concentration through an enzymatic technique (AOAC 986.11) [23]. The evaluation of total and free phosphorus content was conducted using an ascorbic acid molybdate color reagent. Following incubation and measurement of absorbance at 655 nm using a UV–vis spectrophotometer, the results were expressed as g/100 g.

#### 2.2.7. Total Phenol Content

The extract obtained through the process described in the starch section was utilized to determine the total phenolic content. Total phenol was estimated using the FCR assay [25], involving a redox reaction. FCR consists of a combination of tungstates and molybdates, where phenolic compounds in the sample undergo reduction, resulting in the visible formation of a blue color. The absorbance at 650 nm was measured using a UV–vis spectrophotometer, and the results were expressed as gallic acid equivalents (GAE) in g/100 g.

#### 2.2.8. Antioxidant Activity

The ferric-reducing antioxidant activity was determined following the method described by Benzie et al. [26] with slight modifications. A properly diluted sample (0.2 mL) was mixed with 0.8 mL of freshly prepared FRAP reagent. The FRAP reagent was composed of a 10:1:1 ratio of 0.3 M of acetate buffer (pH 3.6), 20 mM of ferric chloride solution (FeCl_3_·6H_2_O), and aqueous 10 mM of TPTZ in 40 mM of HCl. The absorbance of the solution was measured at 593 nm after 10 min of incubation at room temperature. A freshly prepared standard solution of ferrous sulfate was used for calibration, and the FRAP activity was expressed in mg/100 g of gallic acid equivalents (GAE).

### 2.3. Statistical Analysis

The nutritional parameter data were subjected to comprehensive statistical analysis. This included Analysis of Variance (ANOVA), descriptive statistics, and calculation of various genetic parameters such as the phenotypic coefficient of variation (PCV), genotypic coefficient of variation (GCV), heritability (H^2^), and genetic advance percent of means (GAM). These analyses were conducted using the ‘TraitStats’ package [27] implemented in RStudio, R v4.2.3 software (https://cran.r-project.org/bin/windows/base/old/4.2.3/, Vienna, Austria) [28]. Correlations (r) among the nutritional parameters were calculated, and scatter plots were illustrated using the ‘GGally’ package [29] in RStudio, R v4.2.3 software [27]. In the correlation phrase, r > 0 indicated a positive correlation, while r < 0 implied a negative correlation at a *p* < 0.05 significance level. Furthermore, scree plots, principal component analysis (PCA), and cluster diagrams (hierarchical clustering) were generated using the ‘Factoextra’ [30] and ‘FactoMineR’ [28] packages in RStudio, R v4.2.3 software. To compare statistical differences among the clusters, Duncan’s Multiple Range Test (DMRT) was applied, and this was performed using the ‘agricolae’ package in RStudio, R v4.2.3 software.

## 3. Results and Discussion

### 3.1. Nutritional Analysis—Descriptive Statistics of V. angularis Genotypes for Different Nutritional Components

To assess the variability in each trait, descriptive statistics of the data were computed and are summarized in Table 1. The results demonstrated a substantial range of values for every trait, indicating considerable variability: moisture (7.6–13.3 g/100 g), ash (1.80–4.21 g/100 g), protein (18.0–23.9 g/100 g), starch (31.1–43.9 g/100 g), total sugar (3.0–8.1 g/100 g), phytate content (0.652–1.43 g/100 g), phenols (0.010–0.589 g/100 g), and antioxidant (11.4–19.6 mg/g). The observed genetic variations among accessions significantly contribute to the diverse nutritional compositions [31]. For each accession, the mean nutritional composition values for all the traits, along with the critical difference, are presented in Table S1.

#### 3.1.1. Moisture Content

The moisture content of adzuki bean genotypes ranged from 7.55 g/100 g (IC341943) to 13.3 g/100 g (EC057459), with a mean value of 9.39 g/100 g (Table 1). These values were significantly higher than the moisture content of the best-performing check, Totru Local, which was 10.51 g/100 g. The moisture content observed in the studied accessions aligns with previous studies, such as 10.2–11.3 g/100 g reported by Bhatt et al. [32] and 13.1 g/100 g reported by Gohara et al. [33]. The hygroscopic capacity and moisture content of adzuki beans play a crucial role in their cooking characteristics [34]. The lower moisture content is believed to slow down bean hydration during soaking and prolong cooking durations [35]. One popular dish made from adzuki beans is ‘ann’, and its organoleptic qualities are influenced by the moisture content of adzuki bean seeds. Reduced moisture content in sweetened ann leads to increased pastiness and reduced cohesiveness, making it less desirable [35,36].

On the contrary, seed moisture is a critical factor influencing seed quality and storage life. Higher moisture content accelerates seed viability deterioration, primarily due to promoting mold growth, hastening aging, causing thermal damage, and increasing the likelihood of insect infestation. Seed moisture is also directly related to various physiological aspects of seed quality [37]. Therefore, seed moisture is a significant parameter affecting both viability and cooking quality. Based on our study, the top five best-performing accessions for moisture content were identified as EC057459 (13.24 g/100 g), HPKAB87 (12.94 g/100 g), SMLAB4 (g/100 g), EC000264 (11.99 g/100 g), and EC87896 (11.98 g/100 g).

#### 3.1.2. Ash Content

The ash content of adzuki bean genotypes ranged from 1.80 g/100 g (EC340259) to 4.20 g/100 g (IC360533), with a mean value of 2.40 g/100 g (Table 1). The best-performing check, Totru Local, had an ash content of 2.5 g/100 g. However, the ash content in our study was lower than the range of 2.0–7.0 g/100 g reported by Yadav et al. [38]. The ash content determined in this study was comparable to the 4.09 g/100 g reported by Moongngarm et al. [39], and similar results were also found by various other researchers [32,33,40]. Variability in ash content could be attributed to differences in genotypes, soil composition, agricultural practices, and the use of fertilizers during cultivation [39]. Previous studies have indicated that the addition of plant ash can reduce cooking time by up to 60% in beans, while also improving sensory acceptability, antioxidant activity, and nutrient quality [41]. Therefore, ash content represents a crucial nutritional fraction of the seeds. In our study, we identified accessions HPKAB87 (4.21 g/100 g), SMLAB7 (4.11 g/100 g), IC341956 (g/100 g), EC30253 (4.07 g/100 g), and EC24102 (3.94 g/100 g) as the top-performing accessions for ash content.

#### 3.1.3. Total Protein Content

The range of variations in protein content exhibited by accessions ranged between 18.0 g/100 g (IC341945) and 23.9 g/100 g (IC341955), with a mean value of 20.9 g/100 g, and best-performing check HPU-51 giving 21.6 g/100 g (Table 1). The protein content of 24 accessions is more than the best-performing check. ANOVA for 100 accessions of adzuki bean also revealed that variability in protein content caused by genotypes was highly significant (2.2558), whereas treatment variations were non-significant (0.53 Ns) (Table 2). The results conformed with the previously reported adzuki bean work which ranged between 18.3 and 23.8 g/100 g [32,33,42,43]. Protein content higher than our study (23.1–27.5 g/100 g) was obtained by 9 genotypes of adzuki beans from Australia [44]. The differences in the data can be ascribed to variability in the adzuki bean genotypes, environment, and differences in techniques used for analysis. The amount of protein, sugar, fat, calcium, and iron content in adzuki beans varies depending on the soil types in which they were grown [35]. Adzuki bean protein content is at par with other pulses like chickpea (21.5 g/100 g), green gram (22.5 g/100 g), black gram (21.9 g/100 g), and peas (20.4 g/100 g) [45]. Additionally, adzuki bean protein also possesses other known health-promoting properties, such as ACE inhibitory activity (a substance that narrows blood vessels) and anti-inflammatory properties [46,47]. Adzuki beans can be integrated with climate resilience, organic farming, multi-cropping, low carbon and water footprint, and sustainable food production systems. Furthermore, the use of adzuki beans can be improved by creating value-added and nutritionally enriched products like adzuki milk, sprouts, etc., particularly for women’s and children’s nutrition. Owing to its multiple benefits, it is possible to use it as a shield against malnutrition and hidden hunger by developing new varieties. For this, germplasm serves as a building block: from our study, high protein content germplasm can be utilized for crop improvement IC341955 (23.9 g/100 g), EC15256 (23.7 g/100 g), HPKAB87 (or IC360533) (23.4 g/100 g), EC59489 (22.96 g/100 g), and IC341943 (22.79 g/100 g).

#### 3.1.4. Total Starch Content

The starch content among adzuki bean genotypes displayed significant variability, ranging from 31.0 g/100 g (IC353547) to 43.9 g/100 g (IC140846), with a mean value of 39.8 g/100 g (Table 1). The best-performing check had a starch content of 40.2 g/100 g. Interestingly, among the studied accessions, the starch content of 49 accessions exceeded that of the best check. These values surpass the 40.0 g/100 g reported by Kim et al. [48]. The variation in grain starch content can be attributed to the diverse genotypes and the physiological state of the seeds [49,50].

In comparison to other legumes, adzuki bean starch content was higher than chickpea (35.7 g/100 g), pigeon pea (38.8 g/100 g), kidney bean (38.6 g/100 g), and green gram (39.2 g/100 g). However, it was lower than cowpea white (46.3 g/100 g), moth bean (46.4 g/100 g), and horse gram (47.9 g/100 g). Despite its high starch content, its utilization in the food industry remains limited. Adzuki bean starch possesses desirable slow-digesting properties due to its semi-crystalline C-type structure, becoming more digestible during boiling or cooking. Hence, it is suitable for an antidiabetic diet [51,52]. Studies suggest that adzuki bean starch may contribute to reducing cholesterol levels [53]. Consuming whole beans may aid in preventing or managing type 2 diabetes mellitus [54,55,56]. Adzuki bean starch is used in creating popular products like ‘Ann’, adzuki paste, biodegradable film, etc. Therefore, adzuki bean starch stands as a valuable source that can support Sustainable Development Goals 2 and 13, aiming for zero hunger and climate action. Accessions IC140846 (43.24 g/100 g), EC120466 (43.09 g/100 g), IC108080 (42.9 g/100 g), and EC000372 (42.88 g/100 g) were identified as promising from our study.

#### 3.1.5. Total Soluble Sugar (TSS)

The total soluble sugar (TSS) content in adzuki bean genotypes exhibited a notable range, varying from 3.02 g/100 g (IC341943) to 8.17 g/100 g (EC59459), with a mean value of 6.13 g/100 g (Table 1). These values surpass the content of the best-performing check, Grams Local2, which measured 7.2 g/100 g. In fact, the TSS content of 16 genotypes exceeded that of the best check. This range is notably higher compared to the reported range of 2.1 to 4.3 g/100 g, with a mean value of 2.2 g/100 g [32,40]. Elevated TSS concentration enhances desiccation tolerance and augments the seeds’ resistance to abiotic stresses, making it an indicator for identifying drought resistance in adzuki beans [57]. TSS also contributes to improved palatability and serves as a source of fermentable sugars. In various forms, fermented pulse-based food products like uttapam, dosa, idly, vada, sepubari, papad, kanji, and masyaura are commonly consumed in India and other developing countries [58,59]. The high TSS content is particularly beneficial for preparing products like youkan, vellam payar sundal, anpan, manju, wagashi, dorayaki, etc., where sugar is a major ingredient [33]. However, minimal research has been conducted on adzuki bean TSS, warranting further exploration in crop improvement programs to align with current marketing needs and explore new opportunities. Notably, accessions EC59459 (7.97 g/100 g), EC30250 (7.77 g/100 g), EC24102 (7.72 g/100 g), IC469174 (7.69 g/100 g), and EC57159 (7.61 g/100 g) were identified as the best-performing accessions for TSS in our study.

#### 3.1.6. Phytic Acid Content

The phytic acid content showed significant variability among different adzuki bean genotypes, ranging from 0.652 g/100 g (IC360533) to 1.47 g/100 g (IC341952), with an average value of 1.21 g/100 g (Table 1). These values were notably lower than the content in the best-performing check, Grams Local2, which measured 1.18 g/100 g. A total of 40 accessions exhibited phytic acid content lower than the best check. Our findings align with reported values of 0.73–1.08 g/100 g by Han et al. [60] and are similar to the study by Bhatt et al. [32], which reported a value of 1.35 g/100 g.

Phytic acid, which chelates vital minerals like iron, magnesium, calcium, and zinc, hindering their absorption and utilization in monogastric animals due to the lack of phytase, can lead to mineral deficiencies [61]. Phytate also downregulates the activities of proteases and amylases, key food digestive enzymes [62], and reduces the glycemic load. Genotypes with low phytate content are desirable for breeding improved cultivars. Our study highlights low-phytate-containing germplasm, such as EC59459 (0.652 g/100 g), IC360533 (0.655 g/100 g), EC340257 (0.816 g/100 g), EC80850 (0.930 g/100 g), and EC000372 (0.995 g/100 g), which can be valuable in a breeding program. The development of varieties with low phytate content can significantly contribute to combating mineral deficiency and achieving nutritional security.

#### 3.1.7. Phenol Content

The total phenolic content of adzuki bean genotypes exhibited significant variation, ranging from 0.264 g/100 g (EC34027) to 0.588 g/100 g (IC341952), with an average value of 0.387 g/100 g (Table 1). Notably, the best-performing check, Totru Local, had a lower phenol content of 0.10 g/100 g compared to the genotypes. Our study’s findings are consistent with previous research by Bhatt et al. [32], which also showed a wide range of phenol content between 0.486 and 0.648 g/100 g.

Previous studies have highlighted the influence of various factors on adzuki bean seed metabolite contents, including phenolic content. These factors include genotypes or cultivars, temperature, light, latitude, and precipitation [63,64]. Polyphenols, which function as metal chelators, can reduce the digestibility of carbohydrates and proteins, as indicated by Hall et al. [50]. Additionally, the polyphenol extract from the seed coat of the adzuki bean possesses scavenging properties for various radicals, demonstrating its potential health benefits [65,66]. Our study has identified accessions with promising phenolic content, including EC34027 (0.265 g/100 g), EC57159 (0.270 g/100 g), HPKAB98 (0.270 g/100 g), IC341955 (0.275 g/100 g), and EC15256 (0.283 g/100 g), for further exploration and utilization in breeding programs aimed at enhancing nutritional quality.

#### 3.1.8. Antioxidant Activity

The ferric-reducing antioxidant activity of adzuki beans exhibited significant variability across genotypes, ranging from 11.4 mg/g GAE (EC340240) to 19.4 mg/g GAE (IC341957), with a mean value of 15.8 mg/g GAE (Table 1). Notably, the best-performing check, Grams Local2, had an antioxidant content of 16.8 mg/g GAE. Remarkably, 31 genotypes showed higher antioxidant activity than the best-performing check.

Adzuki beans, akin to cowpea, broad beans, and mung beans, possess free radicals such as DPPH, ferric-ion-reducing antioxidant power (FRAP), O_2_^−^·, 2,2′-azino-bis(3-e-htylbenzothiazoline-6-sulfonic acid) (ABTS+), and ·OH scavenging ability [67]. Studies have indicated that polyphenols extracted from the seed coats of adzuki beans exhibit superior scavenging abilities for these free radicals compared to vitamin C [65,66]. Additionally, tocopherols and flavonoids, important antioxidant compounds present in adzuki beans, contribute to lowering the risk of cancer, type 2 diabetes, and heart disease, highlighting their nutritional importance [20].

The variations in antioxidant levels among genotypes can be attributed to differences in the chemical composition of adzuki bean seeds, including variations in seed color, size, shape, and overall genotype [68,69]. Our study has identified several accessions with high antioxidant activity, including IC341955 (19.22 mg/g GAE), IC341957 (19.06 mg/g GAE), IC341950 (18.94 mg/g GAE), IC341953 (18.76 mg/g GAE), and EC18256 (18.34 mg/g GAE), warranting further investigation and potential utilization in breeding programs to enhance the antioxidant profile of adzuki beans.

#### 3.1.9. Comparisons among the Indigenous and Exotic Collections of Adzuki Bean Accessions for Nutritional Traits

**Figure 1:**
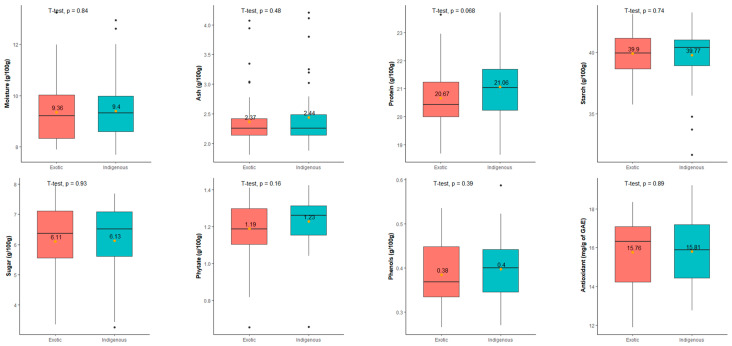
Mean comparison among the indigenous and exotic collections for all the nutrition composition traits. The box plots represent the distribution of traits in the indigenous and exotic groups. The yellow dot with a value in the box plot indicates the mean of the group. The *p*-value indicates the statistical significance (5% level) of the *t*-test.

The comparison of nutritional traits between the indigenous (55) and exotic (45) collections of adzuki bean accessions revealed statistically similar performance across all traits. Figure 1 illustrates the distribution of nutritional values in both groups and employs ‘*t*-test’-based statistical analysis to indicate the significance of differences. Notably, the ‘*t*-test’ results demonstrated non-significant differences for all nutritional traits between the indigenous and exotic collection groups. This suggests a similar pattern of distribution of variation in nutritional composition across both indigenous and exotic collections.

### 3.2. Statistical Analysis

#### 3.2.1. Analysis of Variations (ANOVA) of Nutritional Traits of *V*. *angularis* Accessions

The ANOVA for 100 accessions of adzuki bean is presented in Table 2. It revealed that germplasm accessions showed high variability for all eight nutrition composition traits studied and are highly significant. Our results were inconsistent with previously reported results [59].

#### 3.2.2. Genetic Parameters Analysis for Eight Nutritional Traits for 100 Accessions of *V*. *angularis*

Important genetic parameters like the phenotypic coefficient of variation (PCV), genotypic coefficient of variation (GCV), heritability (H^2^), and genetic advance percent of means (GAM) for nutritional traits are presented in Table 3. Among the nutritional traits, ash exhibited high values for both PCV and GCV, followed by moisture, sugar, phytate, and phenol. Antioxidant content displayed moderate values, while protein and starch showed low values. The heritability (H^2^) was high for all traits, indicating a significant genetic component in their variation. In terms of genetic advance percent of means (GAM), six traits—moisture, ash, sugar, phytate, phenol, and antioxidant content—showed high values. This signifies the presence of substantial additive genetic variance for these traits, making selection based on them an effective and rewarding strategy. On the other hand, protein and starch exhibited low GAM values, suggesting a higher influence of environmental factors on these traits. Consequently, the efficacy of selection for these specific traits may be limited due to the strong environmental impact.

#### 3.2.3. Correlation Analysis of the Nutritional Parameters

**Figure 2:**
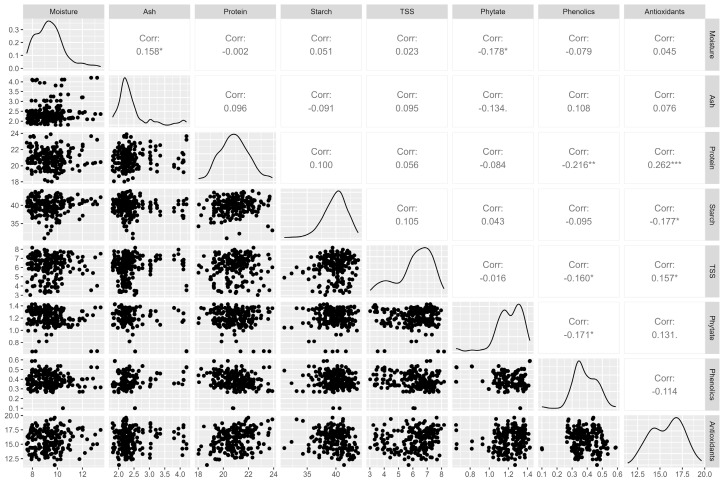
Correlation matrix, distribution plot, and scatter plots among the eight nutritional parameters in *V. angularis.* * *p* < 0.05, ** *p* < 0.01, *** *p* < 0.001.

Correlations among the eight studied traits in 100 genotypes of *V*. *angularis* are presented in Figure 2. These correlations are instrumental in identifying meaningful associations between various parameters.

A highly significant but weak positive correlation was observed between protein and antioxidants (r = 0.262, *p* < 0.01). This suggests that selecting germplasm with high protein content will likely also yield high antioxidant activity, and vice versa. Some plant foods rich in protein tend to have comparable levels of essential amino acids and demonstrate antioxidant activity through electron transfers [70,71]. Protein displayed a significant weak negative correlation with phenols (r = −0.216, *p* < 0.01). Studies on white beans have shown that an increase in phenolic compounds corresponds to a decrease in free amino acids, thiol, and tryptophan residues [72].

Additionally, a significant weak positive correlation was found between antioxidants and TSS (r = 0.157, *p* < 0.05). Legume polysaccharides, with their antioxidant and immunomodulatory properties, have biological activity influenced by sugar content, molecular weight, and glycosidic link types [73]. Conversely, we found a significant weak negative correlation between starch and antioxidants (r = −0.177, *p* < 0.05). Understanding these relationships—between proteins, starch, antioxidants, phytic acid, moisture, ash, TSS, and phenolic compounds—is crucial in food science, as they profoundly impact the functionality of foods [74].

#### 3.2.4. Principal Component Analysis (PCA)

The PCA was a crucial analysis to pinpoint the primary traits responsible for variability and provide a simplified understanding of the data structure. Table 4 outlines the contributions of variables to each principal component. We identified four components, with significant contributions from antioxidant (30.86), phenols (25.38), protein (25.12), ash (33.11), and phytate (30.46) in terms of variable values.

**Figure 3:**
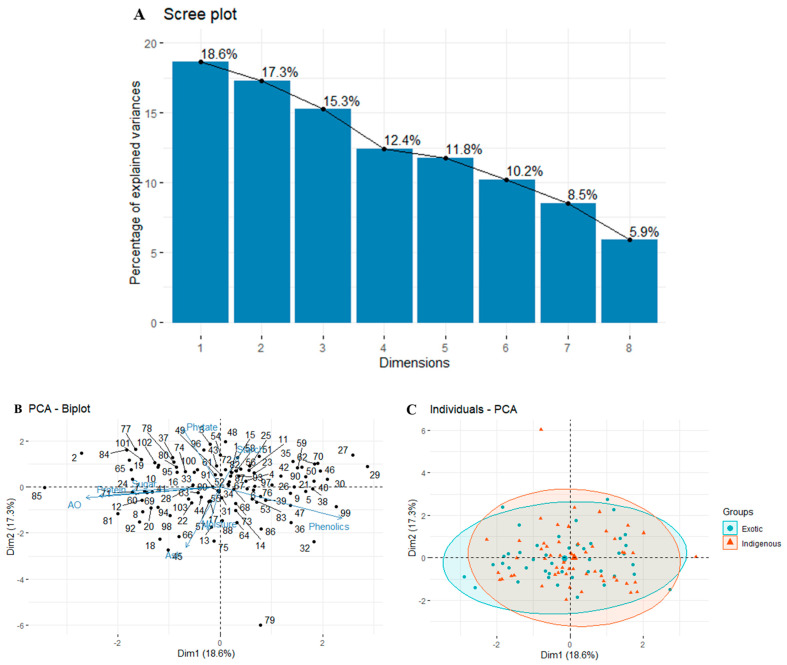
Multivariate analysis among the nutritional traits under study. (**A**) Scree plot explaining principal component variances in terms of percent contribution from each component. (**B**) PCA biplot indicating the distribution of nutritional traits in 100 accessions based on their calculated contribution values for phytic acid, starch, phenols, TSS, protein, antioxidant, moisture, and ash for each principal component. (**C**) Grouping of exotic and indigenous accessions of *V. angularis* based on their nutritional compositions.

Eigenvalues were assigned to each PC, representing a fraction of the dataset’s variation. These eigenvalues were associated with eigenvectors, defining the variation within the main components. To visualize the eigenvalues of the factors or principal components, a scree plot was utilized. Figure 3A showcases the scree plot, presenting principal components based on eigenvalues (>1).

Essentially, the key information was concentrated in the initial principal components (PCs), while the subsequent PCs mainly contained noise [75]. In our study, a significant portion of the total variation (63.6%) was captured by the first four principal components (PC1, PC2, PC3, and PC4) with eigenvalues > 1, as illustrated in Figure 3A. This finding aligns with similar studies conducted on cowpea germplasm accessions, where the remaining principal components contributed minimally to the overall variability [31]. Specifically, PC1 (18.6%) exhibited the most substantial variability across all genotypes, followed by PC2 (17.3%), PC3 (15.3%), and PC4 (12.4%). In total, the first six PCs explained 85.6% of the total variability.

The angles between vectors in Figure 3B signify the associations between traits. A smaller angle denotes a stronger positive correlation, while a 90-degree angle suggests a weaker correlation, and a 180-degree angle indicates a negative correlation. This allowed us to categorize accessions based on their variable relationships and dominant characteristics. To discern differences in nutritional constituents, we categorized exotic and indigenous accessions (Figure 3C). However, the performance in terms of nutritional parameters between exotic and indigenous accessions showed overlapping patterns, and distinct groups did not emerge.

#### 3.2.5. Hierarchical Clustering Analysis

**Figure 4:**
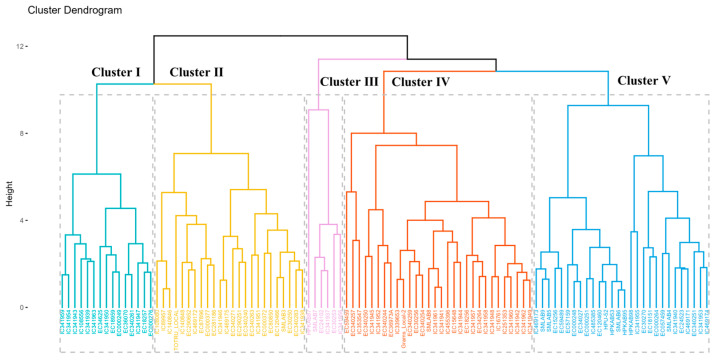
Cluster dendrogram among 100 accessions and three checks of *V. angularis* based on eight nutritional parameters (phytic acid, starch, phenols, TSS, protein, antioxidant, moisture, and ash) Each cluster has been represented with a different color.

Hierarchical clustering analysis (HCA) was utilized to comprehensively assess the multivariate relationships among the nutritional characteristics of the evaluated accessions. This approach elucidates the intricate interconnections between all accessions based on the observed nutritional data, allowing for clustering into distinct groups and facilitating the categorization of these groupings in an unstructured dataset. The cluster dendrogram (Figure 4) delineated the 100 genotypes and three checks into two major clusters. At a distance of 10, these major clusters were further subdivided into five subclusters. Cluster I: This cluster comprised 15 genotypes characterized by low levels of moisture (8.9 g/100 g), ash (2.2 g/100 g), and TSS (4.0 g/100 g) content, with other components present in moderate quantities (Table 5). Cluster II: This included 24 accessions characterized by moderately low levels of all eight parameters. Cluster III: This comprised only six accessions characterized by high ash content (4.2 g/100 g), low phytate (1.15 g/100 g), and moderate levels of other compounds. Notably, accession IC360533, typical of cluster III, exhibited the highest ash (4.21 g/100 g) and lowest phytic acid (0.658 g/100 g) content, making it a desirable trait due to its potential for high mineral bioavailability. Cluster IV: This cluster encompassed the largest number of genotypes (30 accessions) showcasing the lowest starch content (38.1 g/100 g), highest phenol content (0.432 g/100 g), and moderate quantities of other compounds. Cluster V: This encompassed 28 genotypes characterized by the highest moisture (9.8 g/100 g), protein (21.5 g/100 g), TSS (6.7 g/100 g), phytate content (1.246 g/100 g), and antioxidant activity (16.9 mg/g GAE). This cluster displayed the lowest phenol content (0.341 g/100 g) and moderate amounts of ash and starch. Furthermore, a pairwise comparison of all subcluster means through Duncan’s Multiple Range Test (DMRT) indicated significant differences among them for most biochemical attributes. This suggests that accessions from significantly distinct groups can be selected to exploit heterosis effectively, thereby enhancing trait values through plant breeding.

## 4. Conclusions

Our study establishes the presence of significant variations in eight nutritional traits across the adzuki bean accessions preserved at the National Gene Bank of India. Notably, accessions IC341955 (23.7 g/100 g), EC15256 (23.6 g/100 g), and HPKAB87 (23.4 g/100 g) exhibited high protein content. Accessions EC59459 (0.656 g/100 g), HPKAB87 (0.657 g/100 g), and EC340257 (0.818 g/100 g) showcased low phytic acid content. Additionally, accessions IC341955 (19.2 mg/g GAE), IC341957 (19.1 mg/g GAE), and IC341953 (18.7 mg/g GAE) demonstrated high antioxidant activity, establishing them as elite accessions. These promising accessions can serve as parental candidates for augmenting the nutritional profile of adzuki beans in crop improvement programs.

Moreover, accessions displaying favorable agronomic traits and superior nutrient compositions can be further selected, paving the way for the development of enhanced varieties after extensive multilocation and multiseason trials. Nutrient-dense accessions were identified through comprehensive multivariate analysis, particularly hierarchical cluster analysis (HCA). Accessions from cluster III were found to be rich in minerals while also exhibiting low phytate content, whereas cluster V accessions were rich in protein.

Our next steps involve conducting in-depth nutrient profiling encompassing amino acids, sugars, oligosaccharides, minerals, polyphenols, and anti-nutritional factors such as protease inhibitors, oxalates, and saponins. Additionally, we will investigate nutrient bioavailability in the identified elite accessions for a more comprehensive understanding.

## Figures and Tables

**Table 1:** Descriptive statistics for eight nutritional parameters in V. angularis (DW basis).

Sources	Moisture (%)	Ash (%)	Protein (g/100 g)	Starch (g/100 g)	Sugar (g/100 g)	Phytate (g/100 g)	Phenols (g/100 g)	Antioxidant (mg/g GAE)
N	206	206	206	206	206	206	206	206
Mean	9.39	2.40	20.9	39.8	6.13	1.21	0.39	15.9
Minimum	7.55	1.80	18.0	31.0	3.02	0.65	0.10	11.4
Maximum	13.5	4.21	23.9	43.9	8.17	1.43	0.59	19.6
^1^ SE at 5%	0.23	0.01	0.47	0.53	0.19	0.00	0.01	0.39
^2^ CV (%)	3.39	0.11	2.95	1.87	4.36	0.14	2.63	3.63
^3^ CD at 5%	0.63	0.01	1.22	1.48	0.53	0.00	0.02	1.18

^1^ SE represents Standard Error; ^2^ CV represents Coefficient of variance; ^3^ CD represents Critical difference; N represents the total number of germplasms including checks DW represents dry weight.

**Table 2:** Analysis of variances (ANOVA) for eight nutritional parameters in V. angularis.

Source of Variations	df	Moisture (g/100 g)	Ash (g/100 g)	Protein (g/100 g)	Starch (g/100 g)	Sugar (g/100 g)	Phytate (g/100 g)	Phenols (g/100 g)	Antioxidant (mg/g of GAE)
Replications	2	2	2	2	2	2	2	2	2
Genotypes	103	103	103	103	103	103	103	103	103
MSS of replication	1	0.03	0.00 *	0.53	0.22	0.15	0.00 **	0.00	0.44
MSS of genotypes	102	2.64 ***	0.52 ***	2.26 ***	8.51 ***	2.88 ***	0.04 ***	0.01 ***	5.94 ***
Error	102	0.10	0.00	0.38	0.56	0.07	0.00	1 × 10^−4^	0.33
CD		0.63	0.005	1.22	1.48	0.53	0.003	0.0	1.18

MSS represents the mean sum of squares; CD critical difference at 95%; * *p* < 0.05, ** *p* < 0.01, *** *p* < 0.001.

**Table 3:** Genetic parameters analysis for eight nutritional traits for 100 accessions of *V. angularis*.

Parameters	GCV (%)	State	PCV (%)	State	H^2^ (%)	State	GAM (%)	State
Moisture	11.99	Moderate	12.46	Moderate	92.61	High	23.78	High
Ash	21.07	High	21.07	High	99.99	High	43.41	High
Protein	4.63	Low	5.49	Low	71.15	High	8.05	Low
Starch	5.01	Low	5.34	Low	87.72	High	9.66	Low
Sugar	19.29	Moderate	19.78	Moderate	95.13	High	38.77	High
Phytate	11.37	Moderate	11.37	Moderate	99.98	High	23.43	High
Phenols	19.42	Moderate	19.60	Moderate	98.20	High	39.65	High
Antioxidant	10.60	Moderate	11.21	Moderate	89.50	High	20.67	High

PCV represents the phenotypic coefficient of variation; GCV represents the genotypic coefficient of variation; H^2^ represents heritability in a broad sense (%); GAM represents the genetic advance percent of means.

**Table 4:** Contributions of variables for each principal component of PCA.

Parameters	PC 1	PC 2	PC 3	PC 4
Moisture	0.27	17.21	14.23	6.44
Ash	2.04	33.11	0.02	1.62
Protein	25.12	0.75	0.68	47.5
Starch	0.54	8.44	45.33	0.58
Sugar	13.32	0.1	7.86	31.27
Phytate	2.43	30.46	6.68	10.88
Phenols	25.38	8.91	8.76	0.11
Antioxidant	30.86	0.98	16.4	1.57

**Table 5:** The number of genotypes and average values of five clusters for eight different nutritional attributes.

Subcluster	Count	Moisture (g/100 g)	Ash (g/100 g)	Protein (g/100 g)	Starch (g/100 g)	TSS (g/100 g)	Phenolics (g/100 g)	Phytate (g/100 g)	Antioxidant (mg/g)
1	15	8.9 ^a^	2.2 ^b^	21.1 ^ab^	40.0 ^b^	4.0 ^b^	0.396 ^ab^	1.24 ^a^	15.8 ^a^
2	24	9.3 ^a^	2.3 ^b^	20.6 ^b^	41.2 ^a^	6.3 ^a^	0.373 ^b^	1.18 ^a^	13.7 ^b^
3	6	9.8 ^a^	4.0 ^a^	20.9 ^ab^	39.3 ^bc^	6.3 ^a^	0.408 ^ab^	1.15 ^a^	16.0 ^a^
4	30	9.2 ^a^	2.4 ^b^	20.4 ^b^	38.1 ^c^	6.5 ^a^	0.432 ^a^	1.21 ^a^	16.4 ^a^
5	28	9.8 ^a^	2.3 ^b^	21.5 ^a^	40.3 ^ab^	6.7 ^a^	0.341 ^b^	1.25 ^a^	16.9 ^a^

Small alphabet letters represent the Duncan’s Multiple Range Test (DMRT) significance letters to indicate the statistical differences among the subclusters for each biochemical attribute at a 5% level of significance.

## Data Availability

Data are provided in a Supplementary File.

## Supplementary Materials

The following supporting information can be downloaded at: https://www.mdpi.com/article/10.3390/foods12224159/s1, Table S1: *V. angularis* 100 accessions and three checks name, mean values of all the eight nutritional parameters.
