# Supplementary material for: Nutritional Potential of Adzuki Bean Germplasm and Mining Nutri-Dense Accessions through Multivariate Analysis
Source: Foods. 2023 Nov 17;12(22):4159. doi: 10.3390/foods12224159 (PMC10670495; doi:10.3390/foods12224159)
Supplement: Supplementary file 1 [file foods-12-04159-s001.zip › foods-2584704-supplementary.pdf]

Table S1: The mean values of 100 *Vigna angularis* accessions and three checks, for all the eight nutritional parameters.

No.	Genotypes	Moisture (g/100g)	Ash (g/100g)	Protein (g/100g)	Starch (g/100g)	Sugar (g/100g)	Phytate (g/100g)	Phenols (g/100g)	Antioxidant (mg/g of GAE)
1	EC000372	7.95	2.29	21.23	42.88	6.17	0.995	0.298	14.13
2	EC15256	7.95	2.18	23.64	41.81	7.19	1.299	0.283	17.34
3	EC000251	7.90	1.93	20.00	41.12	6.62	1.321	0.349	16.86
4	EC000377	10.55	1.82	20.94	40.92	5.93	1.088	0.325	13.98
5	EC18959	9.68	2.16	20.36	41.47	3.67	1.157	0.498	16.56
6	EC15648	9.04	3.04	19.95	39.61	6.05	1.316	0.407	14.72
7	EC18256	8.09	3.04	20.98	38.70	6.53	1.263	0.347	18.34
8	EC18151	10.11	3.03	22.69	40.19	5.43	1.240	0.393	17.91
9	EC000249	8.34	2.34	20.95	39.94	4.19	1.132	0.499	16.09
10	EC000248	9.34	1.99	20.97	40.79	7.47	1.188	0.284	16.59
11	EC18257	9.55	2.27	19.79	39.80	4.46	1.345	0.380	16.69
12	EC34264	9.44	2.38	22.16	38.48	7.14	1.139	0.359	17.25
13	EC30253	8.14	4.07	20.99	37.82	6.55	1.138	0.346	13.66
14	EC80850	9.32	2.46	20.39	40.45	7.11	0.930	0.380	15.50
15	EC36070	9.11	2.09	20.44	37.85	3.36	1.411	0.334	16.31
16	EC34027	9.35	2.00	20.68	38.88	7.00	1.163	0.265	15.79
17	EC24523	11.14	2.16	21.61	39.84	5.71	1.173	0.397	16.03
18	EC59459	8.13	2.54	21.88	38.80	7.97	0.654	0.373	17.89
19	EC57159	8.12	1.86	21.29	38.84	7.61	1.096	0.270	17.73
20	EC24102	8.78	3.94	20.28	40.74	7.72	1.326	0.359	17.25
21	EC30250	8.85	2.22	20.19	42.18	7.77	1.189	0.485	12.70
22	EC30256	10.27	2.10	21.01	37.97	6.72	1.105	0.372	16.77
23	EC34625	8.82	2.58	20.29	40.97	3.35	1.378	0.448	17.96
24	EC59489	7.91	2.40	22.96	39.81	7.22	1.184	0.351	16.59
25	EC120466	8.19	2.21	20.88	43.09	6.65	1.159	0.401	14.69
26	EC281186	10.12	2.42	20.21	42.69	6.72	1.097	0.338	12.69
27	EC290251	8.10	2.23	19.11	42.90	4.96	1.289	0.468	13.54
28	EC290652	9.42	2.29	21.70	38.52	7.09	1.172	0.315	13.95
29	EC340240	10.02	2.01	19.11	42.14	5.56	1.271	0.463	11.89
30	EC340244	8.83	2.26	19.80	37.89	3.95	1.339	0.507	14.95
31	EC340254	9.91	2.28	20.22	38.64	6.47	1.202	0.435	17.30
32	EC340257	8.83	2.35	20.44	35.75	6.06	0.818	0.532	14.86
33	EC340259	10.05	1.81	19.67	38.70	6.25	1.273	0.346	18.25
34	EC340261	9.78	2.23	21.05	36.47	3.66	1.347	0.397	16.82
35	EC340271	9.11	1.84	18.69	40.62	6.48	1.200	0.368	14.54
36	EC340263	10.14	2.78	21.46	40.64	6.13	1.098	0.536	13.81
37	EC120460	8.89	1.88	21.60	40.60	6.33	1.272	0.327	16.59
38	EC340283	9.11	2.14	20.11	41.67	5.77	1.056	0.460	14.21
39	EC87896	11.98	2.47	19.74	40.68	6.37	1.162	0.320	12.95

40	EC36973A	9.21	2.31	18.83	37.75	6.09	1.366	0.506	14.94
41	EC057459	13.24	2.36	20.42	41.61	7.31	1.373	0.314	17.08
42	EC340250	10.02	2.15	19.01	35.75	4.85	1.332	0.334	14.24
43	EC000276	8.30	2.20	20.60	39.45	4.74	1.252	0.339	16.90
44	EC340251	11.42	2.18	19.80	42.30	7.30	1.090	0.338	16.99
45	EC30270	10.56	3.35	21.90	37.79	7.13	1.091	0.459	17.21
46	IC341938	7.84	2.26	19.90	40.81	6.19	1.155	0.435	12.76
47	IC341951	9.54	1.97	21.80	37.81	5.79	1.041	0.478	13.11
48	IC89957	8.10	2.22	21.46	42.60	7.16	1.372	0.347	12.94
49	IC251353	8.11	2.38	20.69	37.09	5.98	1.363	0.431	16.44
50	IC341946	9.54	2.12	19.81	42.58	4.48	1.177	0.359	14.04
51	IC108556	9.24	2.03	21.51	41.62	3.43	1.298	0.348	14.51
52	IC341940	9.92	2.13	21.21	42.56	6.33	1.150	0.414	15.88
53	IC341948	9.04	2.65	19.73	39.95	5.89	1.176	0.485	17.51
54	IC140846	8.54	2.37	21.90	43.24	7.02	1.319	0.363	13.10
55	IC140848	9.24	3.02	21.29	39.47	6.52	1.236	0.324	12.94
56	IC341942	8.11	1.99	20.70	39.47	6.71	1.306	0.494	16.66
57	IC341950	9.47	2.18	21.66	40.45	4.24	1.071	0.439	18.94
58	IC341943	7.69	2.52	22.79	38.94	3.25	1.313	0.401	14.71
59	IC341939	7.82	2.14	20.88	41.85	4.19	1.106	0.398	14.22
60	IC341958	8.87	2.79	21.69	39.78	7.29	1.238	0.363	17.26
61	IC341962	9.16	2.13	20.93	39.11	5.97	1.298	0.410	16.54
62	IC341963	8.49	1.98	21.36	40.62	4.26	1.296	0.488	13.50
63	IC341960	8.77	2.36	21.34	37.74	6.17	1.243	0.446	17.44
64	IC341944	9.58	3.25	19.69	39.59	7.14	1.157	0.464	16.55
65	IC469173	8.13	2.37	21.61	40.38	7.18	1.362	0.337	17.95
66	IC341956	8.18	4.11	21.04	36.77	6.01	1.278	0.477	18.14
67	IC341947	9.59	2.37	19.79	39.28	4.01	1.112	0.309	16.43
68	IC341961	9.49	2.31	20.70	38.57	7.55	1.279	0.459	15.09
69	IC341957	9.52	2.24	21.42	39.03	7.53	1.261	0.440	19.06
70	IC341952	8.09	1.88	19.81	38.16	7.09	1.425	0.588	14.41
71	IC341953	10.66	2.14	21.71	40.44	6.62	1.196	0.347	18.76
72	IC469172	9.42	2.20	20.17	38.89	6.53	1.273	0.311	14.43
73	IC469171	10.97	2.52	20.99	40.92	6.95	1.185	0.417	14.44
74	SMLAB6	10.09	2.31	20.59	41.69	6.84	1.302	0.334	16.44
75	SMLAB7	10.46	4.11	19.85	40.76	6.69	1.164	0.393	15.92
76	SMLAB8	9.32	2.17	20.09	39.14	7.37	1.117	0.460	15.35
77	SMLAB9	8.00	2.01	22.59	38.77	6.10	1.381	0.300	16.48
78	HPKAB53	8.93	2.33	21.01	41.59	6.63	1.297	0.309	16.36
79	HPKAB87	12.94	4.21	23.44	41.00	3.78	0.657	0.523	14.64
80	HPKAB95	9.51	2.22	20.57	41.67	6.80	1.297	0.344	17.44
81	HPKAB98	11.89	2.42	21.98	36.65	5.55	1.193	0.270	17.87
82	SMLAB3	8.84	2.52	21.34	41.76	6.73	1.260	0.411	14.44
83	SMLAB4	12.61	2.10	20.30	40.73	5.56	1.248	0.406	15.41
84	SMLAB5	8.11	2.13	22.36	40.79	6.06	1.375	0.313	17.79
85	IC341955	9.29	2.25	23.72	33.71	5.78	1.360	0.275	19.22

86	IC353547	9.10	2.46	20.77	31.65	5.13	1.044	0.424	14.86
87	IC341959	10.03	2.34	22.65	40.96	4.62	1.198	0.334	13.15
88	IC339653	10.07	2.60	19.89	36.47	7.36	1.069	0.387	15.81
89	IC341941	9.94	2.30	21.35	39.74	7.58	1.279	0.459	15.13
90	IC469175	10.07	2.22	19.87	40.68	4.06	1.114	0.359	14.67
91	IC455396	10.25	2.46	18.90	39.79	7.47	1.356	0.435	18.21
92	EC000264	11.99	3.20	22.18	41.01	6.95	1.313	0.400	16.97
93	IC341954	9.09	2.09	21.84	40.73	5.25	1.113	0.333	13.72
94	IC469174	10.99	2.15	21.97	41.47	7.69	1.054	0.382	17.14
95	IC108080	9.64	2.70	22.18	42.90	7.13	1.386	0.315	14.63
96	IC485385	7.82	2.16	20.77	41.72	7.37	1.323	0.373	15.75
97	IC16761	8.66	2.04	20.38	39.79	5.84	1.154	0.479	17.22
98	IC341937	10.09	3.80	20.01	38.77	7.09	1.365	0.356	16.59
99	IC341945	9.74	2.19	18.64	34.76	5.66	1.119	0.443	13.55
100	IC341949	8.66	2.21	21.57	40.66	6.51	1.332	0.438	16.79
101	TOTRU_LOCAL	10.44	2.53	20.78	40.08	6.08	1.325	0.101	14.21
102	HPU-51	9.54	2.12	21.33	39.53	6.52	1.352	0.297	16.57
103	Grams_Local-2	9.61	2.49	20.09	37.12	7.15	1.184	0.345	16.71
CD @5%		0.63	0.005	1.22	1.48	0.53	0.003	0.02	1.14

CD: Critical difference
